# Editorial: Methods and protocols in neurotoxicology

**DOI:** 10.3389/ftox.2022.1031667

**Published:** 2022-10-21

**Authors:** Ellen Fritsche, Marta Barenys, Helena T. Hogberg

**Affiliations:** ^1^ IUF–Leibniz Research Institute for Environmental Medicine, Heinrich Heine University of Düsseldorf, Düsseldorf, Germany; ^2^ GRET, INSA-UB, and Toxicology Unit, Pharmacology, Toxicology, and Therapeutical Chemistry Department, Faculty of Pharmacy, University of Barcelona, Barcelona, Spain; ^3^ National Toxicology Program Interagency Center for the Evaluation of Alternative Toxicological Methods (NICEATM), National Institute of Environmental Health Sciences (NIEHS), National Institutes of Health, Durham, NC, United States

**Keywords:** neurotoxicity, methods, *in vitro*, *in vivo*, IATA, validation, developmental

21st century human toxicology is characterized by a rapid evolvement of new technologies and methodologies leading to the development of new approach methods (NAMs; Fischer et al., 2020; Fritsche et al., 2021). The number of NAMs developed has been strongly on the rise over the last years, as they are not only need-, but also science- and technology-driven (Carmichael et al., 2022). Impressive advances in the fields of stem cell technologies, organoid research, microfluidics, organs-on-a-chip as well as in silico approaches have been achieved (Crofton et al., 2022; Hogberg and Smirnova 2022). As the brain is one of the most complex organs of the human body, neurotoxicity assessment has to take the intricacy of this organ into consideration. Especially the relation between adverse effects on a nerve or glia cell and adversity on the functionality of the brain is a grand challenge (Van Thriel et al., 2012; Perez-Catalan et al., 2021; Crofton et al., 2022). Hence, recent approaches in regulatory (developmental) neurotoxicity (D)NT suggest utilization of integrated approaches to testing and assessment (IATA) that integrates all possible data, i.e. epidemiological, *in silico*, *in vitro* and *in vivo*, for decision-making (Krewski et al., 2020). Especially the mechanistic understanding has moved stronger into the focus of toxicity evaluation for regulatory purposes. As a plethora of methods are currently available, this article selection was initiated to compile a selection of methods and protocols contributing to the field of neurotoxicity. This collection covers articles from *in vitro* and *in vivo* methods, from a variety of species, i.e. human, rat, rabbit and Caenorhabditis elegans (*C. elegans*), and from method optimization to scientific/mechanistic validation. Also *in vitro* to *in vivo* extrapolation and application of data in adverse outcome pathways (AOPs) are addressed.

The first article of Culbreth et al. demonstrates the thorough optimization of a human neural progenitor cell-based *in vitro* model for DNT testing in a 384-well high throughput format using high content image analyses (HCA). Generation of such data including their statistical analyses is crucial for understanding test systems and setting up test methods. The authors systematically studied the effects of the extracellular matrix protein laminin as plate coating, cell number and presence of antibiotics in the medium on a fluorescence readout for cell morphology. They optimized the test system and established positive controls for the test method as a necessary step in the method development process. A focus was laid on the biostatistical data evaluation as this method produces a large number of data points for each single cell. The authors state that this assay is now ready for testing DNT compounds.

The second article (Harry et al.) highlights the need to follow a multi-disciplinary approach to evaluate neurotoxicity. Within this approach, authors discuss pros and cons of using rodent behavioral studies and suggest that the understanding of the origin of differences among laboratories and the adoption of several refinements on how such experiments are designed, conducted, analyzed, interpreted and reported could help minimizing variability and maximizing information, as well as reliability of these tests. Particularly, key aspects about observational batteries, motor assessments, measurement of sensory responses, and learning and memory evaluation, among others, are presented. The challenge of translation between adverse effects at a cellular level and alterations in behavior mentioned above is also addressed by the authors, who claim that the next step to solve it is the integration of information from multiple experimental models within a systems biology approach rather than bringing the field to a dichotomy of *in vivo versus in vitro* models.

The third article (Koch et al.) describes the scientific validation of human Neurosphere assays for DNT evaluation. These assays are included in the Organisation for Economic Co-operation and Development (OECD) guidance document (Crofton and Mundy 2021) that instructs on the regulatory use of a DNT *in vitro* battery (IVB). The *in vitro* assays are assessing several neurodevelopmental key events that were previously identified *in vivo* and have evidence of leading to adverse effects if perturbed. Each assay endpoint was validated on relevance to brain development, morphology of specific cell types, expression of cell-type specific markers, the responses of the neurodevelopmental key events to stimuli and power to predict known DNT compounds. Moreover, the authors identified the developmental windows appropriately covered by Neurosphere assays and windows where other assays are needed. The use of the DNT IVB has potential to enhance current DNT testing approaches, hence the scientific validation is crucial to achieve confidence in the assays.

The next article (Pla et al.) presents 14 *in vivo* and *in vitro* protocols for the evaluation of neurodevelopmental alterations in the rabbit species. The authors state that the rabbit model has higher similarity to human brain development than rodents and critically discuss why the rabbit species is relevant for the evaluation of neurodevelopmental alterations in particular cases. Protocols included cover several endpoints currently required in DNT OECD TG 426 (OECD, 2007), ranging from basic behavior ontogeny to complex operant conditioning or sensory behavior, as well as neurohistopathological evaluations of different cell types at different developmental windows. Besides that, *in vitro* protocols cover the adaptation of the human Neurosphere protocol to generate rabbit neurospheres and to perform a rabbit ‘Neurosphere Assay’ for the assessment of neurodevelopmental adverse effects or mechanistic studies helping to reduce the number of animals needed. This approach also enables *in vitro* to *in vivo* extrapolation, and a particular example on data interpretation combining the results obtained in the 14 tests after prenatal hypoxic conditions is given.

The final article (Sammi et al.) explores morphological and behavioral assessments in the model organism *C. elegans* to support new knowledge and mechanistic validation in the Adverse Outcome Pathway (AOP) framework. The authors focused on cholinergic and dopaminergic neurons as they are especially sensitive to neurodegeneration in the most common neurodegenerative diseases. They demonstrated that *C. elegans* assays can be used to measure several key events (KE) both on cellular and organism level and discuss the homology of human genes in these pathways.

It has been acknowledged that many scientific studies have been difficult to reproduce (Ioannidis, 2005). One identified reason is the incomplete information in material and methods in peer reviewed articles (Menke et al., 2022). This research topic was an attempt to encourage better and standardized reporting of experimental design, performance, and results. The methods and protocols featured here have detailed descriptions with a high prospect of inter-lab transferability and reproducibility. Transparency of potential challenges and pitfalls are also defined. Scientific data is only useful if it can be endorsed. One way of building confidence in NAMs is to make sure the data can be reproduced in the hands of others. In addition to scientific publication, it is advised to also cover NAMs in so-called ToxTemps (Krebs et al., 2019). These are based on the OECD (Organization for Economic Collaboration and Development) Guidance Document (GD) 211 (OECD 2017) and hence fulfill all requirements of GD-211, but also inform more strongly on the test systems used by thoroughly describing the cells, guide the user through the types and details of information required and include acceptance criteria for test elements. Such documents are not only necessary for regulatory acceptance of NAMs, but also strongly aid in counteracting the current reproducibility crisis (Baker, 2016).

## References

[B1] Baker M. (2016). 1, 500 scientists lift the lid on reproducibility. Nature 533, 452–454. 10.1038/533452a 27225100

[B2] Carmichael P. L. Baltazar M. T. Cable S. Cochrane S. Dent M. Li H. (2022). Ready for regulatory use: NAMs and NGRA for chemical safety assurance. ALTEX 39 (3), 359–366. 10.14573/altex.2204281 35796331

[B3] Crofton K. M. Bassan A. Behl M. Chushak Y. G. Fritsche E. Gearhart J. M. (2022). Current status and future directions for a neurotoxicity hazard assessment framework that integrates *in silico* approaches. Comput. Toxicol. 22, 100223. 10.1016/j.comtox.2022.100223 35844258 PMC9281386

[B4] Crofton K. M. Mundy W. R. (2021). External scientific report on the interpretation of data from the developmental neurotoxicity *in vitro* testing assays for use in integrated approaches for testing and assessment. EFSA Support. Publ. 18, 42. EN-6924. 10.2903/sp.efsa.2021.EN-6924

[B5] Fischer I. Milton C. Wallace H. (2020). Toxicity testing is evolving. Toxicol. Res. 9 (2), 67–80. 10.1093/toxres/tfaa011 PMC7233318 32440338

[B6] Fritsche E. Haarmann-Stemmann T. Kapr J. Galanjuk S. Hartmann J. Mertens P. R. (2021). Stem cells for next level toxicity testing in the 21st century. Small 17 (15), e2006252. 10.1002/smll.202006252 33354870

[B7] Hogberg H. T. Smirnova L. (2022). The future of 3D brain cultures in developmental neurotoxicity testing. Front. Toxicol. 4, 808620. 10.3389/ftox.2022.808620 35295222 PMC8915853

[B8] Ioannidis J. P. A. (2005). Why Most published research findings are false. PLoS Med. 2, e124. 10.1371/journal.pmed.0020124 16060722 PMC1182327

[B9] Krebs A. Waldmann T. Wilks M. F. van Vugt-Lussenburg B. M. A. van der Burg B. Terron A. (2019). Template for the description of cell-based toxicological test methods to allow evaluation and regulatory use of the data. ALTEX 36, 682–699. 10.14573/altex.1909271 31658359

[B16] Krewski D. Andersen M. E. Tyshenko M. G. Krishnan K. Hartung T. Boekelheide K. (2020). Toxicity testing in the 21st century: Progress in the past decade and future perspectives. Arch. Toxicol. 94, 1–58. 10.1007/s00204-019-02613-4 31848664

[B10] Kustermann S. Kamp H. Hoeng J. Hewitt P. Herzler M. Hengstler J. G. (2019). Template for the description of cell-based toxicological test methods to allow evaluation and regulatory use of the data. ALTEX 36, 682–699. 10.14573/altex.1909271 31658359

[B11] Menke J. Eckmann P. Ozyurt I. B. Roelandse M. Anderson N. Grethe J. (2022). Establishing institutional scores with the rigor and transparency index: Large-scale analysis of scientific reporting quality. J. Med. Internet Res. 24, e37324. 10.2196/37324 35759334 PMC9274430

[B12] OECD (2017). Guidance document for describing non-guideline *in vitro* test methods. OECD Ser. Test. Assess. n 211, 17. 10.1787/9789264274730-en

[B13] OECD (2007). Test no. 426: Developmental neurotoxicity study, OECD guidelines for the testing of chemicals, section 4. Paris: OECD (accessed October 14, 2022). 10.1787/9789264067394-en

[B14] Perez-Catalan N. A. Doe C. Q. Ackerman S. D. (2021). The role of astrocyte-mediated plasticity in neural circuit development and function. Neural Dev. 16, 1. 10.1186/s13064-020-00151-9 33413602 PMC7789420

[B15] Van Thriel C. Westerink R. H. Beste C. Bale A. S. Lein P. J. Leist M. (2012). Translating neurobehavioural endpoints of developmental neurotoxicity tests into *in vitro* assays and readouts. Neurotoxicology 33 (4), 911–924. 10.1016/j.neuro.2011.10.002 22008243 PMC3288589

